# Antibacterial Properties of Fluoride Releasing Glass lonomer Cements (GICs) and Pit and Fissure Sealants on *Streptococcus Mutans*

**DOI:** 10.5005/jp-journals-10005-1060

**Published:** 2010-08-17

**Authors:** Mahesh Kumar M, Mithun Pai BH, Prashant GM, VV Subba Reddy, Usha Mohan Das, Madura C, Chandu GN

**Affiliations:** 1 Postgraduate Student, Department of Community Dentistry, College of Dental Sciences, Davangere, Karnataka, India; 2 Reader, Department of Community Dentistry, College of Dental Sciences, Davangere, Karnataka, India; 3 Principal, Professor and Head, Department of Pedodontics and Preventive Dentistry, College of Dental Sciences, Davangere Karnataka, India; 4 Principal, VS Dental College and Hospital, Bengaluru, Karnataka, India; 5 Assistant Professor, Department of Dermatology, Venereology and Leprosy, SS Institute of Medical Sciences and Research Center, Da-vangere, Karnataka, India; 6 Professor and Head, Department of Community Dentistry, College of Dental Sciences, Davangere, Karnataka, India

**Keywords:** Antibacterial property, fluoride release, glass ionomer cements, pit and fissure sealants, *Streptococcus mutans.*

## Abstract

*Title:* Antibacterial properties of fluoride releasing glass Ionomer cements (GICs) and pit and fissure sealants on *Streptococcus mutans.*

*Background:* Occlusal pit and fissures are the most susceptible sites for dental caries. The clinical effectiveness of GICs and fissure sealants in preventing caries is well-documented, but there is some concern about bacteria left beneath sealants.

*Objective:* (1) Study the antibacterial activity of GICs and pit and fissure sealants. (2) Compare between these materials.

*Methodology:* GICs (Fuji IX GP and Ketac molar) and pit and fissure sealants (Teethmate-F1 and Helioseal-F ). The strains was grown in the Brain Heart Infusion broth and was incubated anaerobically for 18 hours at 37°C and subcultured in MSB agar.Four wells measuring 5 mm diameter was prepared on each agar plate and the prepared materials was placed and further incubated anaerobically for 48 hours at 37°C. The zone of inhibition was measured.

*Results:* All the materials tested showed antibacterial properties to varying levels except; among pit and fissure sealants it is Teethmate-F which showed more antibacterial property.

*Conclusion:* Teethmate-F1 sealant showed more antibacterial property compared to Fuji IX and Ketac molar. Helioseal-F did not show any antibacterial property.

## INTRODUCTION

Prevalence of dental caries has declined in developed countries, mainly in smooth and interproximal surface, but decay in occlusal pits and fissures still has a high prevalence. *In vivo* and *in vitro* studies have shown that *S. mutans* and to a lesser extent *Streptococcus sobrinus,* are isolated from plaque samples of crowns and roots, and are main factors of dental caries.^1^ The therapeutic procedures used in the treatment of caries do not always eliminate all the microorganisms in the residual tissues. The persisting bacterial presence, together with the lack of a thoroughly hermitic seal between the filling and cavity walls, thus allowing bacterial leakage, may be involved in the development of recurring caries.^2^ Cariogenic microorganisms present in the normal human flora could easily penetrate underlying dentin through such defect. Reducing or preferably preventing such marginal breakdown could reduce the chances of recurrent caries.^3,10^ Fluoride, the pivot of preventive dentistry, continues to be the corner stone of caries prevention programs. The success of fluoride in caries prevention of smooth surfaces has made dental caries primarily a disease of pit and fissures of teeth. Occlusal pit and fissures are the most susceptible sites for the development of dental caries and they occur in areas where prevention is difficult. Although, only 12.5% of all the tooth surfaces are occlusal, these surfaces develop more than 2/3 of total caries experienced by children.^4^ The clinical effectiveness of pit and fissure sealants in preventing occlusal caries has been well-documented, but there is some concern about bacteria left beneath sealants. The sealing of pits and fissures resulted in a reduction of 98% of viable bacteria in carious lesions by seal bacteria from the nutrient supply. The fate of the bacteria is of significance when they are inadvertently sealed by a dental sealant.^1^

## OBJECTIVES

1. To study the antibacterial activity of glass ionomer cements and pit and fissure sealants.
2. To compare between two glass ionomer cements and two pit and fissure sealants.

## METHODOLOGY

The materials used for the study were as follows:

1. Glass ionomer cements (Ketac molar and Fuji IX GP )
2. Pit and fissure sealants (Teethmate-Fland Helioseal-F)

The microorganism *S. mutans* isolated in the strains of Mitis Salivarius Bacitracin agar were used. The strains were grown in the Brain Heart Infusion broth and were incubated anaerobically for 18 hours at 37°C. The strains were grown and subcultured in Mitis Salivarius agar. Four wells was prepared on each agar plate using a standard bore with a diameter of 5 mm. Ten specimens of each material was prepared according to manufacturer’s recommendations with the diameter of 5 mm and height of 3 mm and placed in the Mitis Salivarius Bacitracin agar plates. The agar plate was further incubated anaerobically for 48 hours at 37°C. The antimicrobial property of materials was assessed, which were apparent from the circular zones of bacterial inhibition around each material. The diameter of these zones of bacterial inhibition was measured in millimeters using vernier caliper. Statistical analysis was done using SPSS software. The antibacterial property of *S. mutans* was analyzed by Unpaired t-test and ANOVA followed by Tukey’s post hoc test was used wherever necessary.

## RESULTS

Our results showed that fluoride released by Teethmate-Fl sealant, Fiji IX and Ketac molar cements is able to produce an inhibitory effect against *S. mutans* (Tables 1 to 4) (Graphs I to III).

Table 1 shows mean and standard deviation.

Table 2 shows significant difference between each material.

Table 3 shows significant difference between Ketac molar and Fuji IX GP.

Table 4 shows significant difference between Teethmate-Fl and Helioseal-F.

**Table 1:** **Table 1:** Mean and standard deviation

*Materials*		*N*		*Mean*		*Std. Deviation*	
Ketac molar		10		2.18		0.24	
Fuji IX GP		10		5.50		0.62	
Teethmate-Fl		10		8.43		0.42	
Helioseal-F		10		0.00		0.00	

**Table 2:** **Table 2:** Significance difference between each material

*Materials*		*F-value*		*P-value*		*Significance*		*Significance* * difference*	
Ketac molar		895.97		0.00		HS		Ketac molar and Fuji IX, Teethmate-F, Helioseal-F	
Fuji IX GP								Fuji IX and Teethmate- F, Helioseal-F	
Teethmate- F1								Teethmate-F and Helioseal-F	
Helioseal-F									

HS-Highly significant

**Table 3:** **Table 3:** Significance difference between Ketac molar and Fuji IX GP

*Materials*		*t-value*		*P-value*		*Signifficance*	
Ketac molar		16.01		0.00		HS	
Fuji IX GP							

HS-Highly significant

**Table 4:** **Table 4:** Significance difference between Teethmate-F1 and Helioseal-F

*Materials*		*t-value*		*P-value*		*Significance*	
Teethmate-F1		62.56		0.00		HS	
Helioseal-F							

HS-Highly significant

**Graph I:**
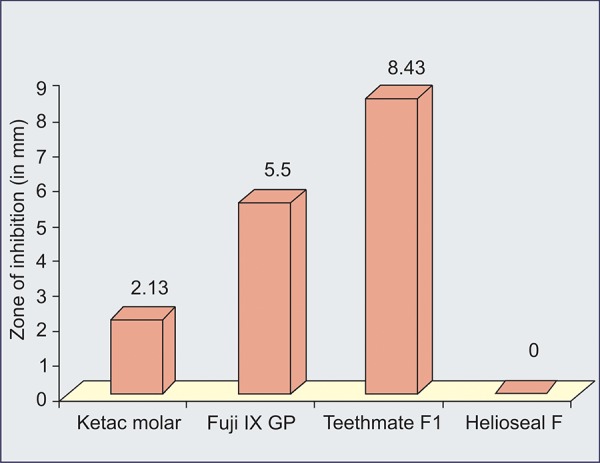
Antibacterial activity of different materials

**Graph II:**
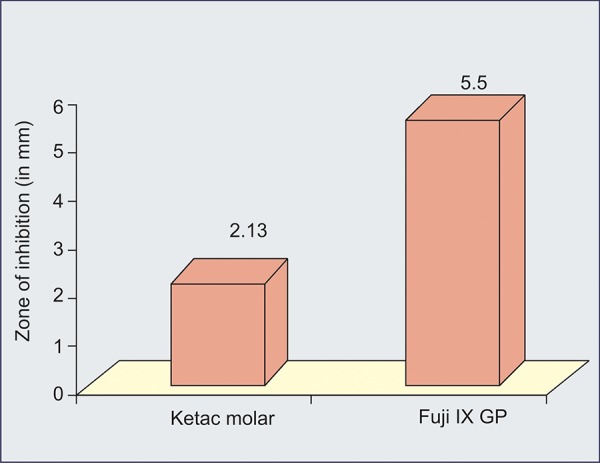
Antibacterial activity between Ketac molar and Fuji IX GP

**Graph III:**
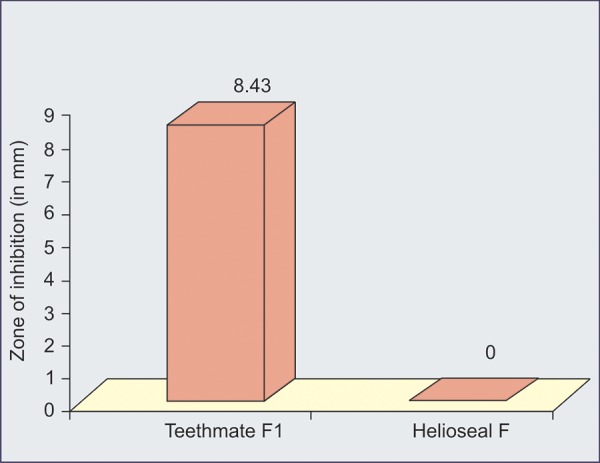
Antibacterial activity between Teethmate-F1 and Helioseal-F

Graph I shows antibacterial activity of different materials.

Graph II shows antibacterial activity between Ketac Molar and Fuji IX GP.

Graph III shows antibacterial activity between Teeth-mate- F1 and Helioseal-F.

## DISCUSSION

Laboratory studies have demonstrated that appreciable amount of fluoride can be released from glass ionomers cements and pit and fissure sealants.^6^ Most clinical studies of pit and fissure sealants agree that a positive relationship exists between the sealant retention and the caries protection benefits, both in preventing and inhibiting the incipient caries in enamel and dentin.^7-9^ During the past several years, the use of sealants has been accepted as an excellent method to prevent dental caries. However, many dentists all over the world do not use sealants for three reasons:

1. Dentist fear that some bacteria could be left within sealed lesions and caries could be progress inadvertently.
2. Sealant application is a time consuming and can treat only a patient at a time.
3. Some dentists consider that there is a better method to prevent dental caries, i.e. fluoridated water in communities.

Present study shows that Teethmate-F1 showed more antibacterial property than Fuji IX GP and Ketac molar. Helioseal-F did not show any antibacterial property.

In a study conducted by Godoy et al, the amount of fluoride released by various fluoridated sealants was measured. Results showed that Teethmate-F1 released significantly more fluoride in comparison to Helioseal-F.^5^ In a similar study conducted by Loyola-Rodriguez and Garcia-Godoy, Teethmate-F1^1^ was the only material that showed antibacterial activity against several strains of *S. mutans* and *S. sobrinus.*

Going et al^4^ have demonstrated that sealant treatment resulted in 89% reversal from the caries-active state to a caries-inactive state. The acid etching procedure itself reduces the number of cultivable microorganism by approximately 95%. Result of the present study showed that fluoride released by the glass ionomer cements and pit and fissure sealant is able to produce an inhibitory effect against *S. mutans.*

These three factors, etching, sealing and antibacterial activity of fluoride, are together almost a guarantee to inhibit the cariogenic bacteria under the glass ionomer cements and pit and fissure sealant.

## CONCLUSION

Teethmate-Fl was the only sealant which showed more antibacterial property when compared to Fuji IX and Ketac molar. Helioseal-F did not show any antibacterial property. The inhibitory effect may have been due to the fluoride released by these materials. All dentists should prefer to use pit and fissure sealant and it is not time consuming. Manufacturer should incorporate antibacterial agents such as chlorhexidine into the cements for better action on microorganism.
